# Family History Extraction From Synthetic Clinical Narratives Using Natural Language Processing: Overview and Evaluation of a Challenge Data Set and Solutions for the 2019 National NLP Clinical Challenges (n2c2)/Open Health Natural Language Processing (OHNLP) Competition

**DOI:** 10.2196/24008

**Published:** 2021-01-27

**Authors:** Feichen Shen, Sijia Liu, Sunyang Fu, Yanshan Wang, Sam Henry, Ozlem Uzuner, Hongfang Liu

**Affiliations:** 1 Division of Digital Health Sciences Mayo Clinic Rochester, MN United States; 2 Department of Information Sciences and Technology George Mason University Fairfax, VA United States; 3 Department of Biomedical Informatics Massachusetts Institute of Technology Cambridge, MA United States; 4 Department of Biomedical Informatics Harvard Medical School Boston, MA United States

**Keywords:** family history extraction, information extraction, natural language processing, named entity recognition, relation extraction

## Abstract

**Background:**

As a risk factor for many diseases, family history (FH) captures both shared genetic variations and living environments among family members. Though there are several systems focusing on FH extraction using natural language processing (NLP) techniques, the evaluation protocol of such systems has not been standardized.

**Objective:**

The n2c2/OHNLP (National NLP Clinical Challenges/Open Health Natural Language Processing) 2019 FH extraction task aims to encourage the community efforts on a standard evaluation and system development on FH extraction from synthetic clinical narratives.

**Methods:**

We organized the first BioCreative/OHNLP FH extraction shared task in 2018. We continued the shared task in 2019 in collaboration with the n2c2 and OHNLP consortium, and organized the 2019 n2c2/OHNLP FH extraction track. The shared task comprises 2 subtasks. Subtask 1 focuses on identifying family member entities and clinical observations (diseases), and subtask 2 expects the association of the living status, side of the family, and clinical observations with family members to be extracted. Subtask 2 is an end-to-end task which is based on the result of subtask 1. We manually curated the first deidentified clinical narrative from FH sections of clinical notes at Mayo Clinic Rochester, the content of which is highly relevant to patients’ FH.

**Results:**

A total of 17 teams from all over the world participated in the n2c2/OHNLP FH extraction shared task, where 38 runs were submitted for subtask 1 and 21 runs were submitted for subtask 2. For subtask 1, the top 3 runs were generated by Harbin Institute of Technology, ezDI, Inc., and The Medical University of South Carolina with F1 scores of 0.8745, 0.8225, and 0.8130, respectively. For subtask 2, the top 3 runs were from Harbin Institute of Technology, ezDI, Inc., and University of Florida with F1 scores of 0.681, 0.6586, and 0.6544, respectively. The workshop was held in conjunction with the AMIA 2019 Fall Symposium.

**Conclusions:**

A wide variety of methods were used by different teams in both tasks, such as Bidirectional Encoder Representations from Transformers, convolutional neural network, bidirectional long short-term memory, conditional random field, support vector machine, and rule-based strategies. System performances show that relation extraction from FH is a more challenging task when compared to entity identification task.

## Introduction

As the key element for precision medicine, family history (FH) captures shared genetic variations and environmental factors among family members [1,2]. Family member demographic information such as age, gender, and degree of relatives is usually taken into account when considering the risk assignment of a large number of common diseases. For example, the risk assessment of hypertrophic cardiomyopathy considers 1 or more first-degree relatives with a history of sudden cardiac death under age 40 as a significant factor of sudden cardiac death risk in patients with hypertrophic cardiomyopathy [3].

Although FH information was largely leveraged to assist the decision-making process of diagnosis and treatment in clinical settings, it remains a challenge to acquire accurate and complete FH information from unstructured text via natural language processing (NLP) methods. FH and negation detection are listed as important attributes in clinical information extraction [4]. One of the major sources of FH data is patient-provided information questionnaires, which are usually stored in a semistructured/unstructured format in electronic health records [5]. In order to provide comprehensive patient-provided FH data to physicians, there is a need for NLP systems that are able to extract FH from the text. Some of the FH data depend on pieces of information provided by patients about their relatives’ health situation during visits. The FH elements may include disease, family member, cause, medication, age of onset of diagnosis, length of disease, etc. This variety of FH elements makes the extraction process from unstructured data challenging.

Although the application of NLP methods and resources to biomedical texts has received increasing attention [6-8], with methods for FH extraction [9-11], the progress has been limited by difficulties in accessing shared tools and resources, partially caused by patient privacy and data confidentiality constraints. There are some recent efforts to increase the sharing and interoperability of existing resources. For example, Azab et al [12] have developed a data set and a baseline system consisting of narrative answers annotated with family histories from FH questionnaires [12], which is based on patient-provided information. The Fast Healthcare Interoperability Resources has also included FamilyMemberHistory as part of the clinical summary standard [13]. To address this issue, we organized this shared task to encourage the community to propose and develop FH extraction systems. Leveraging the research in corpus analysis and deidentification, the Open Health Natural Language Processing (OHNLP) consortium has created multiple deidentified data sets for a couple of NLP tasks based on real clinical sentences [14-16]. In this document, we describe the data set generated for FH extraction from unstructured data. The corpus could be accessed in [17].

## Methods

### Data Preparation

The patient notes we used to curate the corpus were randomly sampled from the Mayo Employee and Community Health cohort. We extracted the section entitled “Family History” in this corpus as the first stage of text selection, and the document structure is presented based on that of clinical notes in Mayo electronic health record according to the CDA R1 (Clinical Document Architecture, Release One) standard [18] without the need for section detection. Then, we have excluded automatically generated semistructured texts because we expected the methods for extracting information from auto-populated formats to be significantly different from extracting information from clinical narratives written by human authors, with the former requiring more engineering effort than NLP research. We have also excluded sections that combine the patients’ social history with the FH section, as these have more descriptions of patients’ personal social behavior such as occupations and life styles instead of family members. As a result, the clinical texts in the corpus focus on narrative patient FH information.

We annotated the corpus using Anafora, a web-based annotation tool for texts [19]. A total of 11 people were involved in the annotation process. Each document is annotated by 2 annotators, and the whole annotation process is performed by a 5-member annotator team (see the “Acknowledgments” section). Thus, there are 10 (2 combinations of 5) distinct pairs of annotators when calculating interannotator agreement (IAA). One senior study coordinator worked as the adjudicator to resolve discrepancies between the 2 annotations.

**Figure 1:**
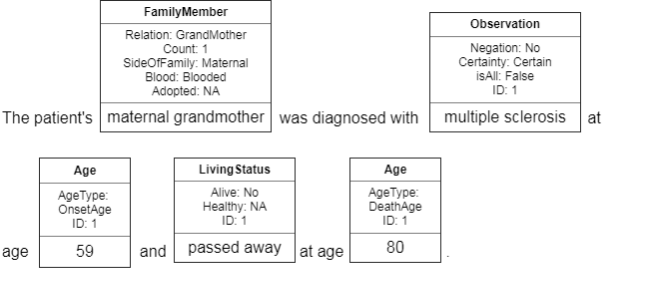
Example entity annotation in FH extraction corpus.

**Figure 2:**
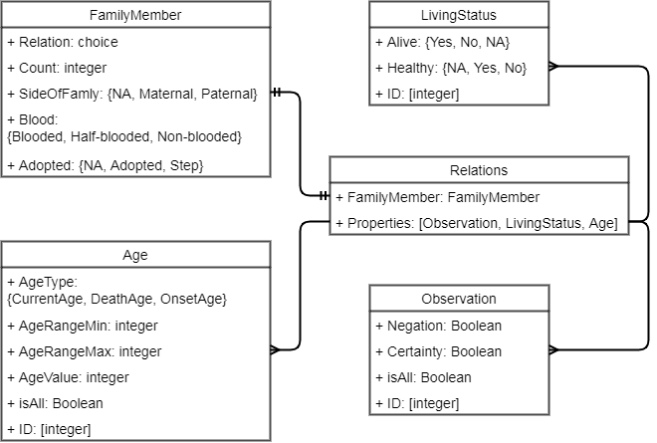
FH extraction annotation schema.

An example of the entity annotation is shown in Figure 1. The sentence “the patient’s maternal grandmother was diagnosed with multiple sclerosis at age 59 and passed away at age 80” is annotated with entities of family members, observation, living status, and ages. The incremental ID field of entities is used to distinguish multiple individuals. In this example, we only have 1 individual under the family member of “maternal grandmother,” so all the IDs are 1. The annotation schema of the FH extraction corpus is illustrated in Figure 2. The corpus is annotated with the following entities and attributes.

#### Family Members

In this study, we annotated only first and second relatives by blood. The spouses were not considered blood relatives, and thus were excluded from the annotation.

Each family member has several properties:

- Side of Family (maternal or paternal): family side mentions are also included in the family member entity annotations.
- Count: the total number of family members under the family member category.
- Blood: whether the family members are fully blood related. For instance, a stepsister with shared mother of the patient is considered “half-blooded.” The default value is “NA” and it applies to most of the family member mentions.
- Adopted: whether the family members are adopted to the family.

#### Observation

This includes any health-related problem including diseases, smoking, suicide, and drinking, excluding auto accident, surgery, and medications. The observation entities have several attributes: negation, certainty, whether the observation applies to all family members, and an integer identifier of family member in case there are more than 1 person in that family category. The negated observations will have a negation field value of “Yes.”

#### Age

The age mentions related to family member, observation, or death are annotated. The word “age” is not annotated in the age mentions. For ranges of age such as “80s,” range min and max values are also annotated.

#### Living Status

Living status are the words and phrases which show health status of the family members. The default value is “Alive: yes” and “Healthy: NA.”

All the entities related to a family member category are linked into 1 chain. In the example shown in Figure 1, the chain has family member of maternal grandmother, and the rest of the chain links other entities related to the family member category. If the patient has multiple family members in the same category (eg, several brothers), all the entities related to any of the brothers will be linked into a chain of “Brother.” The entities can be later restored to each individual family member by their IDs. The incremental IDs are annotated to identify observation, age, and living status from different individuals within the same category.

As part of the annotation process, the data set is manually deidentified with all the patient-protected information, such as names, locations, and age above 89, removed according to the Safety Harbor guideline of Health Insurance Portability and Accountability Act of 1996 (HIPAA) Privacy Rule [20]. To further protect the confidentiality, the observations, family members, and ethnicities are also shuffled among the whole corpus. The numeric fields such as dates and phone numbers are manually replaced with synthetic strings. As a result, the corpus should only be used for studies of information extraction purposes for which the clinical relevance of conditions is not required.

A total of 99 documents for training and 117 documents for testing were included in the released data set. The training set was released to participants and contained both text and annotation files, while for the test set only the raw text files were released. Some statistics on the corpus are listed in Table 1.

**Table 1:** Corpus statistics.

Corpus attribute	Train	Test
Document	99	117
Family member	803	760
Age	757	667
Living status	415	391
Observations	978	1062
Relations	665	631

### Evaluation

For the entity identification subtask (subtask 1), the participants are expected to provide 2 types of information: family members mentioned in the text and the observations (diseases) in the FH. We only used normalized family members for evaluation. The normalized family members are listed in Table 2.

**Table 2:** Normalized family members.

Degree of family members	Normalized family members
1	Father, Mother, Parent, Sister, Brother, Daughter, Son, Child
2	Grandmother, Grandfather, Grandparent, Cousin, Sibling, Aunt, Uncle

In this study, to reduce ambiguities in phrases, we only evaluated if the existence of each family member and mention spans are not taken into account. For family member entities appearing multiple times in a document, only 1 true positive is counted. Regarding the degree of relatives, the side of family should always be “NA” for first-degree relatives (eg, parents, children, siblings).

For the observation mentions, partial matching of the observations is accepted. For example, an extraction of “diabetes” in the phrase “type 2 diabetes” will be considered a true positive when calculating F1 score. We limited the submissions of observations to no more than 4 tokens to avoid abuses of the flexibility.

In subtask 2, the participants need to provide summarized information between family members and observations. For family members, the participants are asked to provide a tuple of (family member, side of family, living status coding). For the observation extraction, the systems are asked to provide a tuple of (family member, side of family, observation). In cases where there are more than 1 observation for 1 family member category, separate tuples are expected.

We used only 1 score to represent living status for each family member category. The patients may have multiple relatives under the family member category (eg, the patient has more than 1 maternal aunts) and sometimes the information provided in the texts was not sufficient for us to analyze. To simplify the comparison in such cases, we encoded the 2 fields of living status (alive and healthy) into 1 integer. For both “Alive” and “Healthy” properties, the results of “Yes,” “NA,” and “No” were encoded as 2, 1, and 0, respectively. The living status score is the alive score multiplied by the healthy score. For example, for a family member with “Alive” as “Yes” and “Healthy” as “Yes,” the living status score should be 2 × 2 = 4. For a family member with “Alive” as “No” and “Healthy” as “NA,” the living status score should be 0 × 1 = 0. Therefore, the higher the encoded living status value, the better the family member’s current condition.

Slightly different from the FH extraction task in 2018, in this year’s challenge, the participants need to detect negation for observations. Specifically, “Negated” and “Non_Negated” should be labeled after each observation.

To be considered as a correct prediction (true positive) for family members, all of the fields have to be matched, including living status. For subtask 2, the observation matching criterion is the same as subtask 1, where partial matching is allowed. Observations applied to all relatives should not be included. For example, in the sentence “there were no reports of mental illness,” the observation of “mental illness” should not appear in any family member entities.

We use standard F1 score as the evaluation (ranking) metrics. Specifically,

Precision = TP/(TP + FP)

Recall = TP/(TP + FN)

F1 = (2 Precision × Recall)/(Precision + Recall)

where true positive (TP) denotes the number of correct predictions, false positive (FP) denotes the number of system predictions that do not exist in the gold standard, and false negative (FN) denotes the number of gold-standard records that do not exist in the system predictions. More details on the evaluation and the evaluation script can be found in [21]. The IAA between 2 annotators measured before the deidentification process in F1 scores was 0.8324 and 0.7002 for subtasks 1 and 2, respectively.

## Results

### Participation

Participating teams were required to sign a data use agreement form to get access to the challenge data set. Each team can submit up to 3 runs for the testing data where each run should have 1 line for each sentence pair that provides the similarity score assigned by the system as a floating-point number. In summary, 41 teams from 7 countries signed up for this shared task; 17 teams submitted 38 systems for subtask 1 (35 of them were valid) and 9 teams submitted 21 systems (20 of them were valid) for subtask 2. Table 3 shows the details of teams that submitted systems, including team names, affiliations, and number of submitted systems.

**Table 3:** Participating teams, affiliations, and the number of submitted systems.

Team	Subtask 1: Entity Identification	Subtask 2: Relation Extraction
Harbin Institute of Technology (HIT)	3	3
ezDI, Inc. (EZDI)	3	3
The Medical University of South Carolina (MUSC)	3	3
National Taitung University (NTTU)	3	N/A^a^
University of Florida (UF)	3	3
Arizona State University (ASU)	3	N/A
The University of Melbourne (MELBOURNE)	2	2
CSIRO Data61 (CSIRO)	1	N/A
University of Aveiro (AVEIRO)	2	2
Dalian University of Technology (DUT)	2	N/A
Yunnan University (YNU)	2	N/A
University of Alabama at Birmingham (ALABAMA)	3	N/A
Med Data Quest: MDQ (MEDDATAQUEST)	3	3
University of Utah (UTAH)	1	1
NED University of Engineering &Technology (NED)	1	N/A
Amrita Vishwa Vidyapeetham (AMRITAVISHMA)	2	N/A
Dalian University of Technology (DUT2)	1	1
Total	38	21

^a^Means the team did not submit their runs for the particular subtask.

### System Performance and Rankings

Tables 4 and 5 list the overall performance of all the valid submitted systems for subtasks 1 and 2, respectively.

For subtask 1, we analyzed IAA for each family member entity and for the entire observation group. From the results shown in Table 6, we found that daughter yielded the optimal F1 score of 1. Father, grandfather, grandmother, sister, mother, and aunt also had high F1 scores. Son was not detected so well, and had the lowest F1 score (0.5926).

Similarly, we also analyzed IAA for subtask 2 as shown in Table 7.

Table 8 lists the top 10 teams with their best runs for subtask 1. The optimal performance was achieved by Harbin Institute of Technology with an F1 score of 0.8745, and the suboptimal performance was yielded by the system built by ezDI, Inc.

For subtask 2, we received fewer submissions and the performance of top 5 systems are shown in Table 9. The system developed by Harbin Institute of Technology performed the best on relation extraction. We observed that errors in the entity extraction tasks will pass on to the relation extraction task, causing errors in predicting the observations and family member living status. Second, from previous studies on end-to-end relation extraction tasks, the performance in relation extraction tasks is lower than that in named entity recognition tasks [22,23]. A successful system also needs to consider co-reference resolution, which could be considered a standalone task for NLP systems [24].

**Table 4:** Overall performance for subtask 1.

Statistic	F1 score (n2c2/OHNLP^a^ family history extraction 2019 subtask 1)
Max	0.8750
Min	0.0000
Median	0.7341
Mean	0.7659
SD	0.1472

^a^n2c2/OHNLP: National NLP Clinical Challenges/Open Health Natural Language Processing.

**Table 5:** Overall performance for subtask 2.

Statistic	F1 score (n2c2/OHNLP^a^ family history extraction 2019 subtask 2)
Max	0.6810
Min	0.2241
Median	0.5616
Mean	0.6222
SD	0.1247

^a^n2c2/OHNLP: National NLP Clinical Challenges/Open Health Natural Language Processing.

**Table 6:** Interannotator agreement for subtask 1.

Family member	Precision	Recall	F1	Instance count
Daughter	1	1	1	58
Father	0.9636	0.9464	0.9550	160
Grandfather	0.9429	0.9429	0.9429	111
Grandmother	0.9302	0.9524	0.9412	130
Sister	0.8462	1	0.9167	116
Mother	0.92	0.8846	0.9020	170
Aunt	0.8889	0.9143	0.9014	131
Uncle	0.9063	0.8529	0.8788	112
Grandparent	1	0.7143	0.8333	13
Brother	0.7941	0.8182	0.8060	105
Cousin	0.8333	0.75	0.7895	90
Observation	0.8478	0.6536	0.7382	1913
Parent	0.7143	0.7143	0.7143	10
Child	1	0.5	0.6667	15
Sibling	0.6667	0.6667	0.6667	19
Son	0.6154	0.5714	0.5926	65

**Table 7:** Interannotator agreement for subtask 2.

Family member	Precision	Recall	F1	Instance count
Son	1	1	1	65
Brother	0.85	0.8947	0.8718	105
Grandfather	0.8649	0.8649	0.8649	111
Cousin	0.7692	0.9091	0.8333	90
Grandmother	0.7333	0.8462	0.7857	130
Uncle	0.7917	0.7308	0.76	112
Aunt	0.7429	0.7027	0.7222	131
Grandparent	0.6667	0.6667	0.6667	13
Mother	0.5349	0.7302	0.6174	170
Father	0.5775	0.5395	0.5578	160
Sister	0.5161	0.5517	0.5333	116

**Table 8:** Performance of the top 10 teams for subtask 1.

Rank	Team	Precision	Recall	F1
1	Harbin Institute of Technology (HIT)	0.9154	0.8372	0.8745
2	ezDI, Inc. (EZDI)	0.8090	0.8365	0.8225
3	The Medical University of South Carolina (MUSC)	0.7890	0.8384	0.8130
4	National Taitung University (NTTU)	0.8043	0.8093	0.8068
5	University of Florida (UF)	0.7969	0.7920	0.7944
6	Arizona State University (ASU)	0.7655	0.8105	0.7874
7	The University of Melbourne (MELBOURNE)	0.7327	0.8111	0.7699
8	CSIRO Data61 (CSIRO)	0.7048	0.8322	0.7632
9	University of Aveiro (AVEIRO)	0.6501	0.8892	0.7510
10	Dalian University of Technology (DUT)	0.8690	0.6533	0.7458

**Table 9:** Performance of the top 5 teams in subtask 2.

Rank	Team	Precision	Recall	F1
1	Harbin Institute of Technology (HIT)	0.7459	0.6265	0.6810
2	ezDI, Inc. (EZDI)	0.6999	0.6220	0.6586
3	University of Florida (UF)	0.6995	0.6184	0.6544
4	The Medical University of South Carolina (MUSC)	0.6548	0.6441	0.6494
5	University of Aveiro (AVEIRO)	0.5703	0.525	0.5467

### Methods Description

The list of techniques used by each team for subtask 1 is shown in Table 10. We found that many teams used the state-of-the-art NLP contextual neural language models in their systems, such as Bidirectional Encoder Representations from Transformers (BERT) [25] and ELMo [26]. We also observed that deep learning architecture with pretrained embeddings was widely used by many teams. Besides these, 4 teams incorporated rule-based strategy into their systems for entity identification.

**Table 10:** Techniques used in the top systems for subtask 1.

Team	Techniques
Harbin Institute of Technology (HIT)	BERT^a^ + CNN^b^ for character features, MLP^c^, biaffine classifier
ezDI, Inc. (EZDI)	Deep learning + rule-based approach
The Medical University of South Carolina (MUSC)	Bi-LSTM^d^ + character level CNN + CRF^e^ with ELMo representations, voting ensemble method
National Taitung University (NTTU)	Bi-LSTM + CRF, UMLS^f^ embedding
University of Florida (UF)	RCNN^g^ + BERT
Arizona State University (ASU)	BIO tagging + BERT
The University of Melbourne (MELBOURNE)	ELMo embedding + Bi-LSTM
CSIRO Data61 (CSIRO)	Bi-LSTM + CRF with ELMo representations for observations, rule-based for family member
University of Aveiro (AVEIRO)	Dependency parsing + co-reference + rule-based
Dalian University of Technology (DUT)	Rule-based + dictionary-based

^a^BERT: Bidirectional Encoder Representations from Transformers.

^b^CNN: convolutional neural network.

^c^MLP: multilayer perceptron.

^d^Bi-LSTM: bidirectional long short-term memory.

^e^CRF: conditional random field.

^f^UMLS: Unified Medical Language System.

^g^RCNN: region-based convolutional neural networks.

Brief descriptions of the techniques used by the top 5 teams that submitted methodology for subtask 2 are listed in Table 11. Similar to techniques used for subtask 1, we found that the ensemble of BERT, deep learning architecture, and some other conventional machine learning algorithms are common strategies adopted by different teams. In addition, rule-based approaches were used in some submissions with BERT and NLP techniques for relation extraction.

**Table 11:** Techniques used in the top 5 systems for subtask 2.

Team	Techniques
Harbin Institute of Technology (HIT)	BERT^a^ + CNN^b^ for character features, MLP^c^, biaffine classifier
ezDI, Inc. (EZDI)	Support vector machine
University of Florida (UF)	Rule-based + BERT
The Medical University of South Carolina (MUSC)	Vowpal Wabbit library for relation classification + FastContext for negation detection
University of Aveiro (AVEIRO)	Dependency parsing + co-reference + rule-based

^a^BERT: Bidirectional Encoder Representations from Transformers.

^b^CNN: convolutional neural network.

^c^MLP: multilayer perceptron.

## Discussion

### Study Limitations

We have conducted an error analysis over common mistakes made by different systems. For detecting family member, the most common error was found in the step of co-reference resolution. For example, one document states “Paternal family history is positive for Leo himself speculating he may have had ADHD that was never diagnosed or treated. Owen’s son (Samuel's paternal cousin) has been diagnosed with Asperger syndrome.” Leo is the patient here and Owen’s son is not Leo’s paternal cousin. However, some systems recognized such paternal cousin mention as the Leo’s cousin incorrectly. In another example, the document states that “Mike’s sister (Kate’s paternal aunt) has a history of being exceedingly smart, but she always got poor grades.” Some systems did extract sister as a correct mention, but paternal aunt was also extracted as a false-positive case. All the names that appeared in the above examples are synthetic.

For observation, we roughly categorized the common mistakes into 2 groups. The first group is related to annotation disagreement or errors made by annotators. In Anafora, it is required for human annotators to select the span of the word/phrase and annotate them as different type of entities. Taking breast cancer as an example, some annotators selected the whole phrase as 1 annotation, but some others only selected the span for “breast” and “cancer” but overlooked the space in between. Similarly, taking “suicides” as an example, some annotators only selected the span to cover the word “suicide” but did not annotate “s,” but some other did. There also exist some disagreements regarding inferred semantic meaning of a specific observation. For example, some annotators annotated “Struggled with math” and “keeping a job” as observations but some did not. The second group is related to errors made by the participants’ systems. We observed that most of such errors occurred due to false positives, indicating that those observations/conditions are beyond first or second degree. In the first example above, Owen’s son was diagnosed with Asperger syndrome and he has no blood relationship with the patient Leo. But some systems extracted Asperger syndrome as the observation incorrectly.

In the future work, we will give an updated training session to the annotators with the lesson learned from this task, in order to make uniform annotation criteria as well as improve annotation agreement. In addition, we plan to increase the number of FH cases coming from different institutions. Moreover, we will add more entities and attributes in the evaluation.

### Conclusions

We summarize the 2019 n2c2/OHNLP FH extraction shared task in this overview. In this task, we have developed a corpus using deidentified FH data stored in Mayo Clinic. The corpus we prepared along with the shared task has encouraged participants internationally to develop FH extraction systems for understanding clinical narratives. We compared the performance of valid systems on 2 subtasks: entity identification and relation extraction. The optimal F1 score for subtask 1 and subtask 2 is 0.8745 and 0.6810, respectively. We also observed that most of the typical errors made by the submitted systems are related to co-reference resolution. The corpus could be viewed as valuable resources for more researchers to improve systems for FH analysis.

## Abbreviations

AMIA: American Medical Informatics Association
BERT: Bidirectional Encoder Representations from Transformers
Bi-LSTM: bidirectional long short-term memory
CNN: convolutional neural network
CRF: conditional random field
FH: family history
MLP: multilayer perceptron
n2c2: National NLP Clinical Challenges
NLP: natural language processing
OHNLP: Open Health Natural Language Processing
RCNN: region-based convolutional neural networks
UMLS: Unified Medical Language System
